# Pleiotropic effects of rosuvastatin on the glucose metabolism and the subcutaneous and visceral adipose tissue behavior in C57Bl/6 mice

**DOI:** 10.1186/1758-5996-5-32

**Published:** 2013-07-01

**Authors:** Rodrigo Neto-Ferreira, Vinícius Novaes Rocha, Vanessa Souza-Mello, Carlos Alberto Mandarim-de-Lacerda, Jorge José de Carvalho

**Affiliations:** 1 Laboratory of Ultrastructure and Tecidual Biology, Biomedical Center, Institute of Biology, State University of Rio de Janeiro, 20551-030, Rio de Janeiro, RJ, Brazil; 2 Laboratory of Morphometry and Cardiovascular Morphology, Biomedical Center, Institute of Biology, State University of Rio de Janeiro, Rio de Janeiro, Brazil

**Keywords:** Rosuvastatin, Insulin resistance, Adipose tissue, NAFLD

## Abstract

The aim of this study was to evaluate whether rosuvastatin (HMG-CoA reductase inhibitor) modulates the carbohydrate and lipid metabolism, the development of non-alcoholic fatty liver disease (NAFLD), and the increase in body mass in a model of diet-induced obesity. Male C57Bl/6 mice (3-months-old) were fed a high-fat diet (HF, 60% lipids) or the standard chow (SC, 10% lipids) for 15 weeks. The animals were then treated with 10 mg/kg/day (HF-R10 group), 20 mg/kg/day (HF-R20), or 40 mg/kg/day (HF-R40) of rosuvastatin for five weeks. The HF diet led to glucose intolerance, insulin resistance, weight gain, increased visceral adiposity with adipocyte hypertrophy, and hepatic steatosis (micro and macrovesicular). The rosuvastatin treatment decreased the adiposity and the adipocyte size in the HF-R10 and HF-R20 groups. In addition, rosuvastatin changed the pattern of fat distribution in the HF-R40 group because more fat was stored subcutaneously than in visceral depots. This redistribution improved the fasting glucose and the glucose intolerance. Rosuvastatin also improved the liver morphology and ultrastructure in a dose-dependent manner. In conclusion, rosuvastatin exerts pleiotropic effects through a dose-dependent improvement of glucose intolerance, insulin sensitivity and NAFLD and changes the fat distribution from visceral to subcutaneous fat depots in a mouse model of diet-induced obesity.

## Background

Non-alcoholic fatty liver disease (NAFLD) is the most common cause of chronic liver disease and encompasses a number of diseases, from steatosis (lipid deposition) and non-alcoholic steatohepatitis (NASH, inflammation) to cirrhosis (fibrosis) and liver failure [1]. It affects up to 20% of the population in Western countries [2] and is commonly found in patients with visceral obesity, insulin resistance, dyslipidemia and hypertension [3]. Anatomically, steatosis can take one of two forms depending on the size of the lipid vesicles: microvesicular steatosis is the condition in which fat is stored in multiple small vesicles within the hepatocyte cytoplasm, whereas macrovesicular steatosis refers to the condition in which fat is stored in a single large vesicle [4]. The exact physiopathology of NAFLD and especially the factors that lead to the progression from steatosis to steatohepatitis and end-stage liver disease are not fully understood [3]. Promising treatments for NASH include antioxidants, hepatoprotective agents, antidiabetic drugs, such as insulin sensitizers, and lipid-lowering agents [5,6]. Rosuvastatin is a lipid-lowering agent that competitively inhibits the 3-hydroxy-3-methylglutaryl coenzyme A (HMG-CoA) reductase. Rosuvastatin exhibits the highest efficacy in the reduction of LDL cholesterol, total cholesterol and triglycerides compared with other statins at comparable doses [7]. It has been shown that rosuvastatin has an increased number of binding sites to the HMG-CoA reductase enzyme compared with other statins, which would explain its stronger inhibition capability and thus its greater therapeutic efficacy. Moreover, rosuvastatin has been found to be a highly effective hypolipidemic agent in patients with metabolic syndrome [8]. In addition, rosuvastatin reduces the risk of cardiovascular disease, decrease vascular reactive oxygen species generation independently of cholesterol reduction [9] and exerts several “pleiotropic”, but also achieve significant improvement in endothelial function [10] effects that may result in a further clinical benefit [11,12].

Recent studies have shown that rosuvastatin ameliorates hepatic insulin resistance in rodents and humans [13,14]. Even with some concern in relation to safety, the use of statins to tackle NAFLD has been encouraged by “ National Lipid Association ” [15], mainly considering positive results from important trials such as JUPITER in which patients at high risk to new diabetes onset also benefited from rosuvastatin use [16]. However, studies evaluating the use of statins to treat insulin resistance and NAFLD are scarce in the literature.

Given the increasing prevalence of obesity, the research community is seeking experimental models that both mimic the human phenotype and are suitable for testing potential therapies to treat metabolic syndrome (MS) and other associated metabolic disorders [17]. C57Bl/6 mice fed a high-fat (HF) diet are a useful experimental model for studying MS, as has been shown in previous studies [18,19]. Thus, the aim of the present study was to evaluate whether rosuvastatin modulates the carbohydrate and lipid metabolism, the level of non-alcoholic fatty liver disease (NAFLD), and the body mass gain in a model of diet-induced obesity.

## Methods

### Experimental groups

All of the procedures were conducted in accordance with the conventional guidelines for animal experimentation (NIH Publication No. 85–23, revised in 1996). All of the experimental protocols were approved by the animal ethics committee of the State University of Rio de Janeiro. The animals were housed under controlled conditions (temperature at 21±2°C, humidity at 60±10% and a 12-h/12-h dark/light cycle) and had free access to food and water. The mineral and vitamin contents of the two diets were identical and were consistent with the American Institute of Nutrition’s recommendation (AIN 93M) [20]. The mouse chow was prepared by Pragsolucoes (Jau, São Paulo, Brazil).

At baseline, after one week of acclimatization, 50 three-month-old C57Bl/6 male mice were randomly divided and fed different diets during a 15-week period, which included a SC diet (standard chow; 10% lipids, n=10) or a HF diet (60% lipids, n=40), both diets are detailed in Table 1. The 15 week period of administration of HF diet aimed at inducing the main features of the metabolic syndrome. Before being divided into two groups, the homoscedasticity of variances was tested and all animals followed the normal distribution and had not differences concerning body mass, which guarantee that different groups started the experiment without any difference that could add bias to the study.

**Table 1:** **Composition and energy content of the SC and HF diet I cal** = **4**.**184J**

**Content** (**g**/**kg**)	**Diet**	
	**SC**	**HF**
Casein (≥85% of protein)	140.0	190.0
Cornstarch (g/kg)	620.7	250.7
Sucrose (g/kg)	100.0	100.0
Soya-bean oil (g/kg)	40.0	40.0
Lard (g/kg)		320.0
Fibre (g/kg)	50.0	50.0
Vitamin mix (g/kg)*	10.0	10.0
Mineral mix (g/kg)*	35.0	35.0
L-cystin (g/kg)	1.8	1.8
Choline (g/kg)	2.5	2.5
Antioxidant (g/kg)	0.008	0.008
Total mass (g)	1.000	1.000
Energy content (kcal/kg)	3.573	5.404
Carbohydrates (%)	76	26
Protein (%)	14	14
Lipids (%)	10	60

*Mineral and vitamin mmixtures are in accordance with AIN 93M.

Afterwards, the HF group was randomly divided into 4 groups (n=10 each) in order to begin treatment with rosuvastatin (Crestor; Astrazeneca). Consequently, five groups were formed, as follows: SC group (standard chow during the whole experiment / n=10); HF group (high fat diet during the whole experiment /n=10); HF-R10 group (high fat diet plus rosuvastatin, 10 mg/kg/day / n=10); HF-R20 group (high fat diet plus rosuvastatin, 20 mg/kg/dia / n=10); HF-R40 group (high fat diet plus rosuvastatin, 40 mg/kg/dia / n=10). Treatments lasted for 5 weeks and drug was mixed with the diet. Fresh chow was provided daily, and any remaining chow from the previous day was discarded. The food intake was evaluated daily (1 p.m.), and the body mass was measured weekly. Taking daily food consumption and BM into account, the drug doses were corrected to match the same concentrations as indicated.

The energy contents in the high-fat diet and the standard show were 5.404 kcal/g and 3.573 kcal/g, respectively. The feed efficiency was assessed after 15 weeks of the SC or HF diet intake (pre-treatment) and after treatment (post-treatment) as the energy intake in KJ divided by body mass in g, expressed as KJ/g BM [6].

Concerning the endpoints evaluated, five animals from each group were chosen at random for stereological and blood biochemistry analyses, while the five remaining were used for western blotting and transmission electron microscopy. All analyses were carried out in a blinded fashion.

### Oral glucose tolerance test (OGTT)

To evaluate the glucose tolerance, an OGTT was performed after 15 weeks of diet intake (before treatment) and again five weeks after treatment. The animals fasted for 6 h and then received 25% glucose in sterile saline (0.9% NaCl) at a dose of 1 g/kg by orogastric gavage. The blood was collected through a little incision at the tip of the tail, and the plasma glucose concentration was measured (glucometer; Accu-Chek Active; Roche Applied Science, Brazil). The plasma glucose was assessed before glucose administration and 15, 30, 45 and 120 min after glucose administration. The response was expressed as the area under the curve (AUC) (GraphPad Prism version 5.03; San Diego, CA, USA).

### Blood biochemistry

The animals were food-deprived for 6 h and then deeply anaesthetized with sodium pentobarbital (150 mg/kg i.p.). The blood was sampled by cardiac puncture at the right atrium and then centrifuged (120×g for 15 min) at room temperature. The total cholesterol (TC), triglycerides (TG), and markers of hepatic function, such as aspartate aminotransferase (AST) and alanine aminotransferase (ALT), in the serum were measured by a colorimetric enzymatic assay (Bioclin, Quibasa, Belo Horizonte, MG, Brazil).

The insulin level was determined by radioimmunoassay (cat. no. RI-13K; Linco/Millipore; intra-assay coefficient of variation of 1.4%). The insulin resistance (IR) was estimated through the homeostasis model assessment index (HOMA-IR): (insulin × glucose)/ 22.5 [21].

### Hepatic triglyceride

Liver samples were frozen at −80°C. The level of hepatic triglycerides was measured according to a previously published protocol [22]. Briefly, 50 mg of frozen liver tissue was placed in an ultrasonic processor with 1.0 mL of isopropanol. The homogenate was centrifuged at 2,000 × *g* and 5.0 μl of the supernatant was used. The hepatic triglyceride was then assessed using a colorimetric enzymatic assay (K55, Bioclin, Quibasa, Belo Horizonte, MG, Brazil).

### Liver stereology

Several fragments from all parts of the liver were prepared, included in Paraplast Plus (Sigma Aldrich, St. Louis, MO, USA), sectioned into 3-μm sections and stained with hematoxylin–eosin. Five random microscopic fields were analyzed per animal through the use of video-microscopy (Leica DMRBE microscope with plan achromatic objectives, Leica, Wetzlar, Germany) and a 36-point test-system (PT) [23]. The volume density (V*v*) of steatosis was estimated by point counting the fat droplets in the hepatocytes: V*v* [steatosis] = PP[steatosis] / PT, where PP is the number of points that hit the lipid droplets [24].

### Liver ultrastructure

The liver samples were processed for transmission electron microscopy. Ultra-thin sections (Leica Ultracut ultramicrotome) were counterstained with uranyl acetate and lead citrate and observed with a Zeiss EM 906. The number density of mitochondria (Q_A_) was defined as the number of mitochondrial profiles within a frame with a known area (expressed as the number of mitochondria/μ^2^). A total of 45 electron micrographs was evaluated per group, and all of the mitochondria within the test frame, except those that hit the forbidden lines or their extensions, were counted [25].

### Adipocyte morphometry

After euthanasia, the epididymal (visceral fat) and inguinal (subcutaneous fat) fat pads were collected and weighed, and these values were used to calculate the visceral: subcutaneous (Visc:Sub) fat ratio. Histological slices of the epididymal fat pad were prepared, and digital images were obtained (LC Evolution camera; Olympus BX51 microscope and Media Cybernetics Image-Pro Plus version 7.0; TIFF format; 36-bit color; 1, 280 × 1,024 pixels). The mean cross-sectional area of at least 50 adipocytes per mice was estimated [26].

### Western blot analysis

The total hepatic proteins were extracted in homogenizing buffer with protease inhibitors. The homogenates were then centrifuged at 3200 × *g* and 4°C for 20 min, and the supernatants were collected. Equal quantities of total protein were resuspended in SDS-containing sample buffer, heated for 5 min at 100°C and separated by SDS/PAGE. After electrophoresis, the proteins were electroblotted onto PVDF transfer membranes (Hybond-P; Amersham Biosciences) and visualized with Ponceau solution staining. The membranes were then blocked by incubation in 6% (w/v) non-fat dry milk in TBS-T (Tris-buffered saline [20 mmol/l Tris/HCl pH 7.4 and 500 mmol/l NaCl] with 0.05% Tween-20), incubated with polyclonal antibodies against rabbit SREBP-1 (sterol regulatory element-binding protein-1; 68 kDa; SC-367; Santa Cruz Biotechnology), washed and incubated with anti-rabbit IgG secondary antibody. The SREBP-1 protein expression was detected using an ECL (enhanced chemiluminescence) detection system (Amersham Biosciences). The signals were visualized by autoradiography and quantified through a quantitative analysis of the digital images (Image-Pro Plus version 7.0). The integral absorbance values were measured. The structural b-actin proteins (Santa Cruz Biotechnology, code sc-81178, CA, USA) were obtained by stripping the nitrocellulose membrane proteins of the liver tissue.

### Data analysis

The values are shown as the means ± SEM. In all of the cases in which homoscedasticity among the variances was confirmed, the data were analyzed using ANOVA followed by post-hoc Tukey’s test. If homoscedasticity was not confirmed, the differences were analyzed using the Kruskal-Wallis test and the post-hoc Dunn’s test. A P value ≤ 0.05 was considered statistically significant (GraphPad Prism version 5.03 for Windows).

## Results

### Body mass and food intake

**Figure 1:**
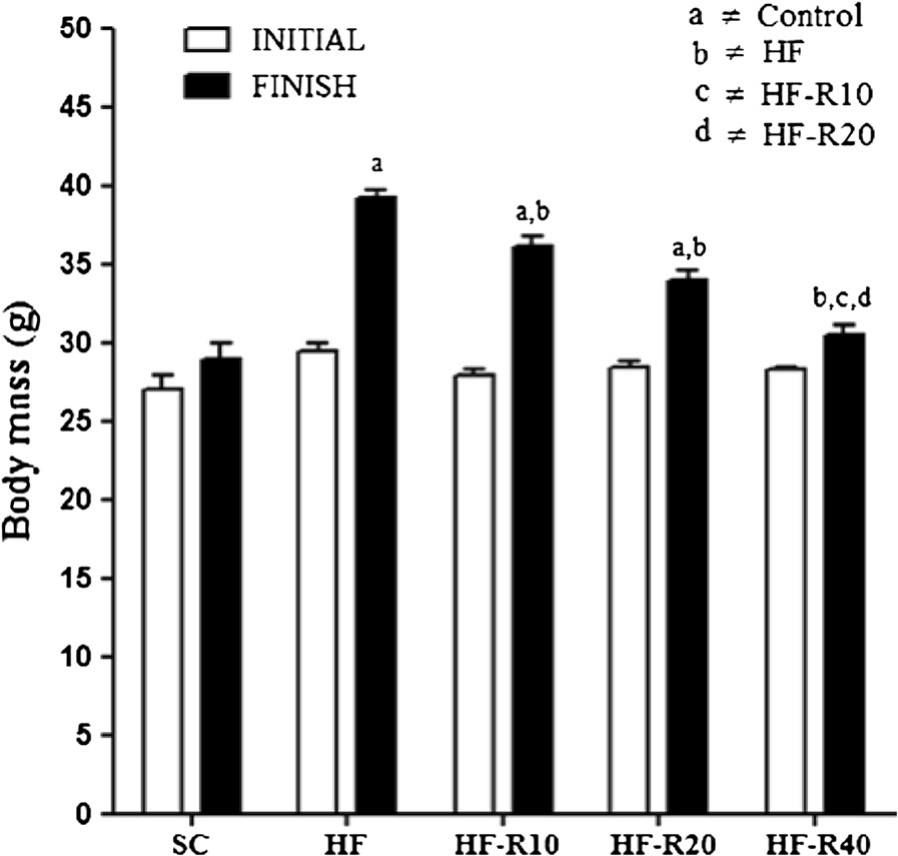
**Initial and finish body mass.** Mice were fed the standard chow (SC) or a high-fat diet (HF) for 15 weeks. The mice then received five weeks of rosuvastatin at doses of 10 mg/kg/day (HF-R10), 20 mg/kg/day (HF-R20), or 40 mg/kg/day (HF-R40). The symbols indicate a difference compared with [a] the SC group, [b] the HF group, [c] the HF-R10 group and [d] the HF-R20 group.

The body mass (BM) of the mice fed the HF diet for 15 weeks increased progressively compared to the animals that received the standard chow (p <0.001, Figure 1). After five weeks of rosuvastatin treatment (20 weeks on the respective diet), the BM remained greater in the HF, HF-R10 and HF-R20 groups compared with the SC group. The 40 mg/kg/day dose of rosuvastatin reduced the body mass gain of the HF-R40 group. The HF-R40 group exhibited a lower BM than the HF, HF-R10 and HF-R20 groups (p <0.001, Figure 1).

After ratifying the body mass gain in the HF group, we found an increased food intake and a higher energy intake in these animals (+23% and +85% respectively, p <0.0001, Table 2). Despite the lower increment in body mass observed in the HF-R40 mice, the administration of rosuvastatin dose-dependently increased the food intake and the ernegy intake in the HF-R10, HF-R20 and HF-R40 groups compared with the untreated HF group. Feed efficiency was evaluated through the ratio KJ/g BM and behaved likewise energy intake, where HF group showed the highest feed efficiency (+30% in comparison to SC, p <0.0001), followed by the rosuvastatin treated animals in a dose dependant manner, being feed efficiency of HF-R40 bigger than HF-R20 and HF-R10 (+22%, p <0.05).

**Table 2:** **Feeding behavior**, **carbohydrate metabolism and blood biochemistry**

**Parameters**	**Groups**				
**Pre**-**treatment**	**SC**	**HF**			
Food intake (g/day)	2.9±0.01	3.7±0.03			
Energy (kJ/day per mouse)	43.4±0.34	83.7±0.64^a^			
OGTT (AUC)	1,174 ±46.55	1,259±45.06^a^			
**Post**-**treatment**	**SC**	**HF**	**HF**-**R10**	**HF**-**R20**	**HF**-**R40**
Food intake (g/day)	2.2±0.01	2.7±0.02 ^a^	2.8±0.02 ^a,b^	2.9±0.02 ^a,b^	3.0±0.01 ^a,b,c,d^
Energy (kJ/day per mouse)	32.6±0.2	60.4±1.1^a^	63.7±0.5^a,b^	64.6±0.4^a,b^	68.5±0.2^a,b,c,d^
Feed efficiency (kJ/g BM)	1.2±0.02	1.6±0.05^a^	1.8±0.07 ^a^	1.8±0.05^a,b^	2.2±0.05 ^a,b,c,d^
Glucose (mg/dL)	109±6.22	149.6±9.9^a^	134.2±10.4	82.4±7.4 ^b,c^	63.8±6.4 ^a,b,c^
Insulin (mU/ml)	5.85±0.95	20.4±2.0^a^	20.3±4.1^a^	16.86±1.20^a^	14.0±0.32
OGTT (AUC)	990.1±37.8	1,264.2±41.8^a^	1,236.8±6.0^a^	1,037.7±34.7^b,c^	859.3±50.4^b,c^
HOMA-IR	1.79±0.23	7.51±0.81^a^	6.66±1.42^a^	6.53±0.39^a^	2.04±0.23^b,c,d^
Serum TC (mg/dL)	103.6±18.1	226.2±16.0^a^	163.0±16.1^b^	128.8±12.3^b^	114.6±7.8^b^
Serum TG (mg/dL)	51.0±4.6	87.1±4.8^a^	82.0±7.8^a^	43.6±8.4^b,c^	50.0±2.2^b,c^
ALT (U/L)	15±2.9	115±15.2^a^	113±19.3^a^	69.4±9.0^a^	62.2±8.4^a,b^
AST (U/L)	272±28.1	289±35.4	283±31.6	191.6±35.5	188.2±33.8

Abbreviations: *SC* standard rodent chow, *HF* high-fat diet, *HF*-*R10* 10 mg/kg/day of rosuvastatin, *HF*-*R20* 20 mg/kg/day of rosuvastatin, *HF*-*R40* 40 mg/kg/day of rosuvastatin, *OGTT* oral glucose tolerance teste, *AUC* area under the curve, *HOMA*-*IR* homeostasis model assessment index for insulin resistance, *ALT* alanine aminotransferase, *AST* aspartate transaminase, *TC* total cholesterol, *TG* triglyceride. Symbols represent difference with: [a] SC group; [b] HF group; [c] HF-R10 group; [d] HF-R20 group.

### Carbohydrate metabolism

The mice from the HF group exhibited glucose intolerance after 15 weeks on the HF diet (pre-treatment, Table 2). Before rosuvastatin treatment, the AUC of the OGTT was higher in the HF mice than in the SC mice (7% higher, p <0.01). This difference was still observed at the end of the experiment. The administration of rosuvastatin at doses of 20 and 40 mg/kg/day improved the glucose intolerance. The AUC of the OGTT was lower in the HF-R20 (−17%, p <0.001) and HF-R40 (−32%, p <0.05) groups compared with the HF group (Table 2).

At the end of the experiment, the HF mice were also insulin-resistant compared with the mice that were fed the standard chow (HOMA-IR was 25% higher in the HF group, p <0.05). The three doses of rosuvastatin improved the insulin sensitivity. The HOMA-IR was 11% lower in the HF-R10 group compared with the HF group (p <0.01) and much lower in the HF-R40 group (−70%) compared with the HF-R10 group (p <0.01, Table 2).

### Blood biochemistry

The total cholesterol (TC) increased in the untreated HF mice compared with the SC group (+119% higher, p <0.01), and the three doses of rosuvastatin reduced the TC (p <0.001, Table 2). The same pattern was observed with the serum triglycerides (TG) (Table 2). The plasma ALT concentration was also significantly elevated in the untreated HF mice compared with the SC (p <0.0001) and HF-R40 groups (p <0.0271). The plasma AST did not differ among groups.

### Adipose tissue

The high-fat diet increased the visceral fat mass (retroperitoneal and epididymal fat pads, Table 3). This fat mass was significantly greater in the HF group (+605%, p <0.001) than in the SC group, and the 10 mg/kg/day and 20 mg/kg/day doses of rosuvastatin did not affect it (+455% and +386% in the HF-R10 and HF-R20 groups, respectively, compared with the SC group, p <0.05). In contrast, the visceral fat mass decreased in the mice treated with 40 mg/kg/day of rosuvastatin (−56% in the HF-R40 group compared with the HF group, p <0.01) to levels similar to those measured in the SC group. The subcutaneous adipose tissue (inguinal fat) was significantly higher in the HF-R10 and HF groups than in the SC group (+312%, p <0.01) (Table 3). In addition, the administration of rosuvastatin changed the pattern of fat distribution. The HF diet induced an increase in the visceral fat, as shown by the Visc:Sub ratio (Table 3). However, rosuvastatin was able to prevent the fat redistribution from subcutaneous to visceral fat depots.

**Table 3:** Adipose tissue weight and morphology

**Parameters**	**Groups**				
	**SC**	**HF**	**HF-R10**	**HF-R20**	**HF-R40**
Visceral fat (mg)	146±26.0	1,030±102.7^a^	811±145.5^a^	710±98.9^a^	455±72.5^b^
Subcutaneous fat (mg)	97±7.0	400±49.5^a^	400±51.1^a^	320±36.9	298±54.9
Visc:Sub ratio	0.74±0.11	0.39±0.03^a^	0.48±0.04^a^	0.50±0.05	0.67±0.05^b^
Adipocyte diameter (μm)	48.6±0.8	101.6±4.6^a^	73.7±3.2^a,b^	66.0±2.3^a,b^	51.5±2.6^b,c,d^

Abbreviations: *SC* standard rodent chow, *HF* high-fat diet, *HF*-*R10* 10 mg/kg/day of rosuvastatin, *HF*-*R20* 20 mg/kg/day of rosuvastatin, *HF*-*R40* 40 mg/kg/day of rosuvastatin, *Sub* subcutaneous fat (=inguinal fat), *Visc* visceral fat (=epididymal plus retroperitoneal fat). Symbols represent difference with: [a] SC group; [b] HF group; [c] HF-R10 group; [d] HF-R20 group.

Hypertrophied adipocytes were observed in the HF group. The adipocyte diameter was 109% higher (p <0.001) in the HF group than in the SC group (Table 3), and the rosuvastatin treatment had a dose-dependent effect on this parameter. Only the HF-R40 group exhibited adipocytes with a diameter similar to those observed in the SC group, whereas the other groups (HF-R10 and HF-R20) had intermediate-sized adipocytes.

### Liver

**Figure 2:**
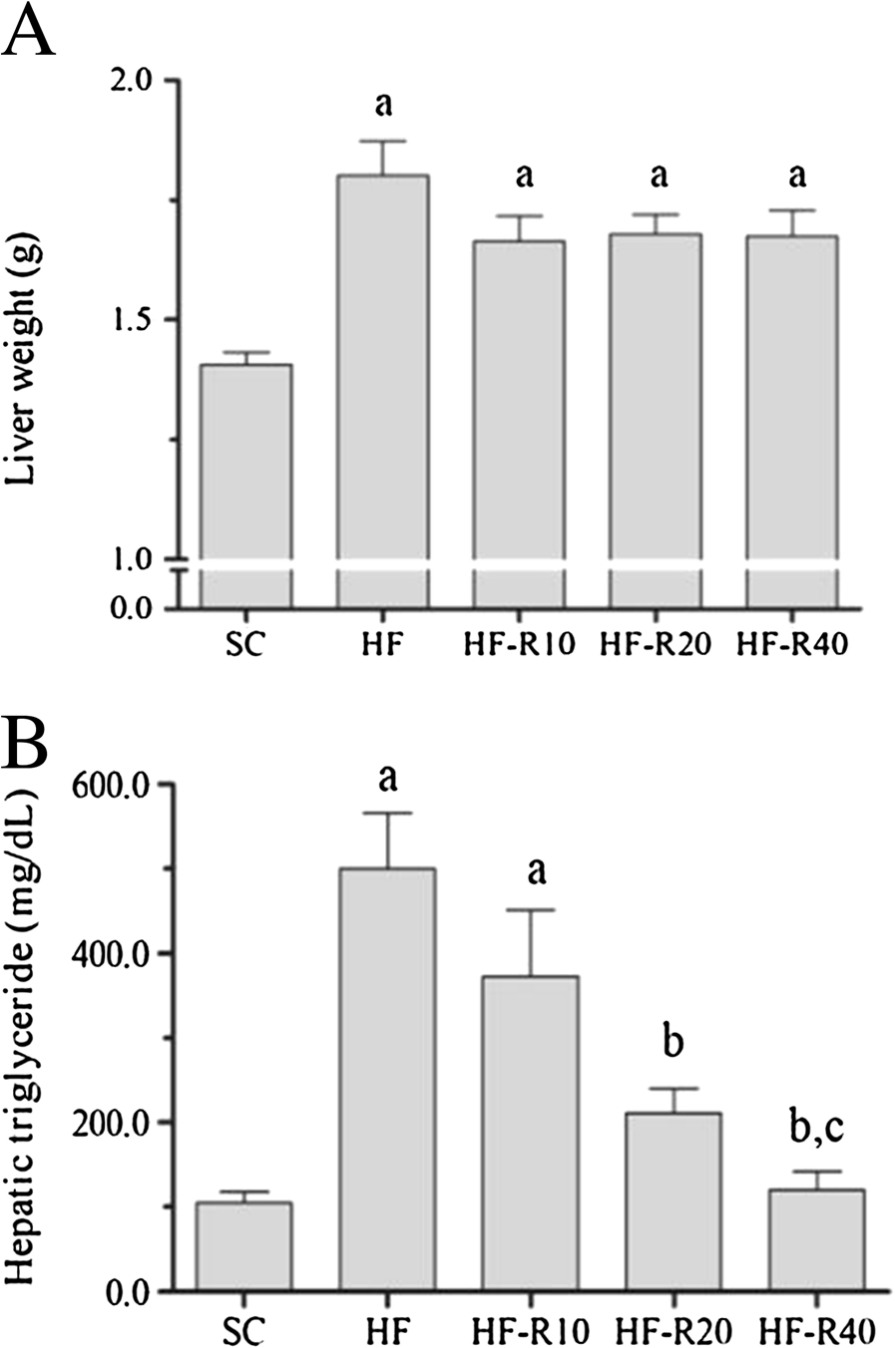
**Pleiotropic effects of rosuvastatin on the liver morphology.** Mice were fed the standard chow (SC) or a high-fat diet (HF) for 15 weeks and then received five weeks of rosuvastatin treatment at doses of 10 mg/kg/day (HF-R10), 20 mg/kg/day (HF-R20), or 40 mg/kg/day (HF-R40). **(A)** Liver weight corrected by tibia length. **(B)** Hepatic triglyceride content. The symbols indicate a difference compared with [a] the SC group, [b] the HF group and [c] the HF-R10 group.

**Figure 3:**
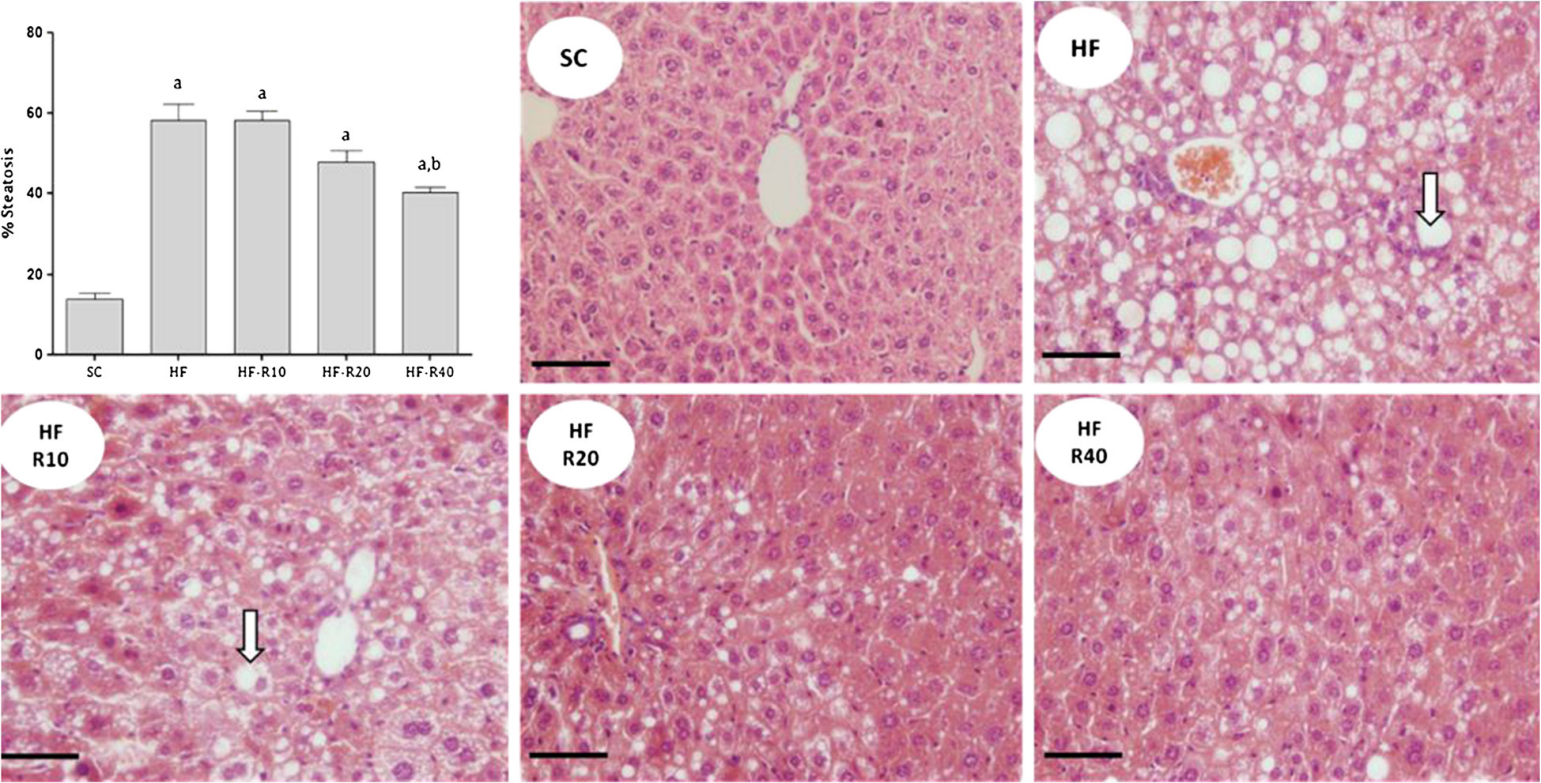
**Liver photomicrographs stained with hematoxylin and eosin (bar = 30 μm).** Note the normal liver morphology in the SC group, whereas the HF groups exhibit micro and macrovesicular steatosis. The rosuvastatin treatment ameliorated the steatosis in a dose-dependent manner, as observed in the treated groups: HF-R10, HF-R20 and HF-R40. The arrows indicate the steatosis. The symbols indicate a difference compared with [a] the SC group, [b] the HF group and [c] the HF-R10 group.

**Figure 4:**
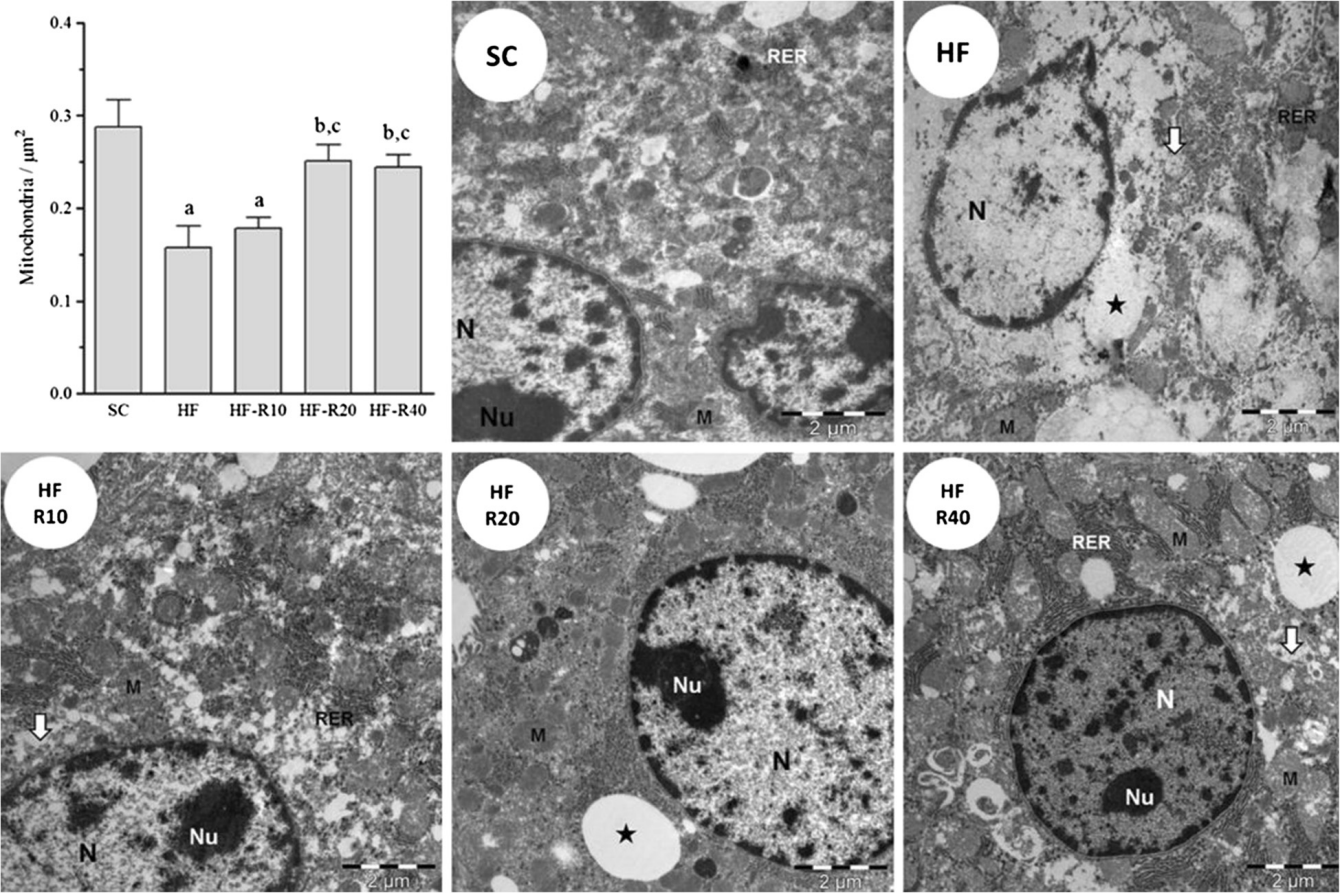
**Pleiotropic effects of rosuvastatin on the liver ultrastructure.** Mice were fed the standard chow (SC) or a high-fat diet (HF) for 15 weeks, followed by five weeks of rosuvastatin treatment at doses of 10 mg/kg/day (HF-R10), 20 mg/kg/day (HF-R20), or 40 mg/kg/day (HF-R40). Numerical density of mitochondria assessed by stereology. Electron micrographs of the liver (bar = 2 μm). The nucleus (N), nucleolus (Nu) and rugous endoplasmic reticulum (RER) are well preserved, and a number of mitochondria (M) are observed in the SC group. The HF mice exhibit severe macrovesicular (star) and microvesicular (arrow) steatosis, unorganized RER and few mitochondria. Microvesicular steatosis is also found in the HF-R10 group. In contrast, the HF-R20 group has a regular cytoarchitecture, a typical number of mitochondria and spread lipid droplets. A regular nucleus, nucleolus and mitochondria, as well as diminished steatosis, are observed in the HF-R40 group. The symbols indicate a difference compared with [a] the SC group, [b] the HF group and [c] the HF-R10 group.

Excessive fat intake led to liver enlargement in all of the groups that received the HF diet compared with the SC group (Figure 2A). The levels of hepatic triglycerides were significantly higher in the HF group (+400%, p = 0.0001) than in the SC group (Figure 2B). The rosuvastatin treatment dose-dependently decreased the hepatic triglycerides in the mice from the HF-R10, HF-R20 and HF-R40 groups compared with the untreated HF group (p <0.0001, Figure 2B). As observed through light microscopy, liver steatosis was recurrent in our study, especially in the HF group, which showed severe microvesicular and macrovesicular steatosis (Figure 3). Although both the HF-R20 and HF-R40 groups showed an attenuation of the hepatic steatosis, only the HF-R40 group was statistically different from the untreated HF group. The electron microscopy also revealed hepatocytes with abundant lipid vesicles in the HF group (Figure 4), which characterizes the existence of macro and microvesicular steatosis and a complete breakdown of the cytoarchitecture. Smaller and scarcer mitochondria were also found. The mitochondrial cristae were difficult to observe, and a remarkable breakdown of the rough endoplasmic reticulum and Golgi apparatus was found (Figure 4). In contrast, the treated groups showed smaller and less frequent fat droplets, which confirms the findings obtained through light microscopy. The untreated HF mice exhibited a lower density of mitochondria compared with the SC (p <0.001), HF-R20 (p <0.001) and HF-R40 (p <0.05) groups (Figure 4).

#### Western blot analysis

**Figure 5:**
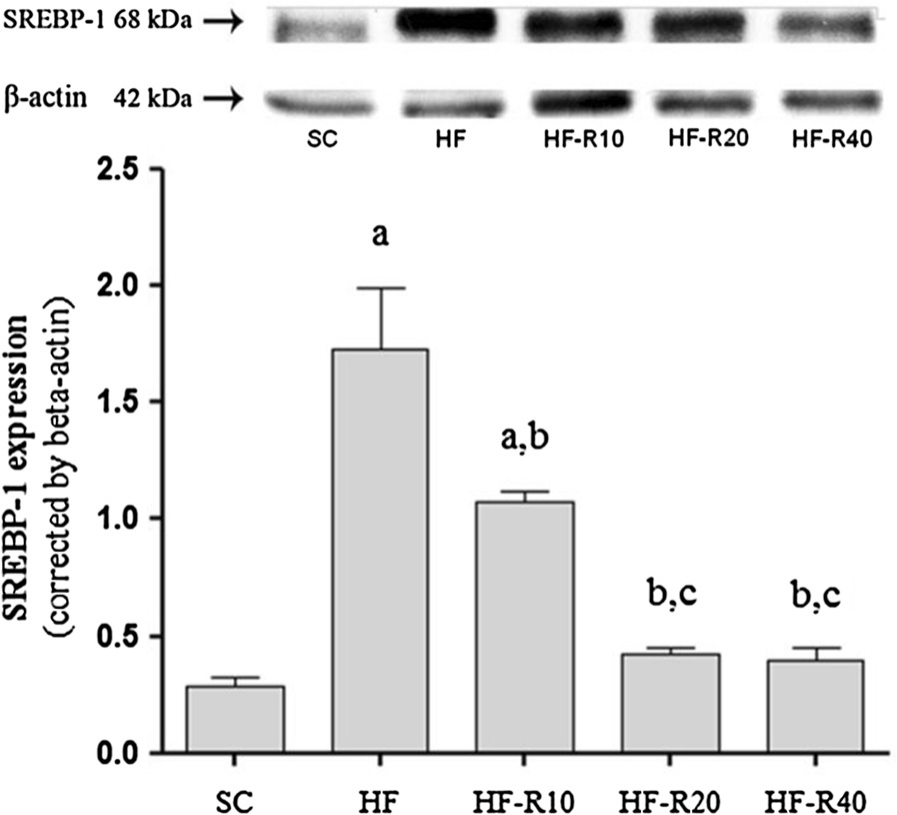
**Western blot analyses of hepatic SREBP**-**1.** A representative immunoblot is shown; the quantification of the bands in the immunoblot is shown below. The values are the means ± S.E.M. from five experiments. The symbols indicate a difference compared with [a] the SC group, [b] the HF group and [c] the HF-R10 group.

The SREBP-1 expression was higher in the untreated HF group compared with the SC group (+614%, *P* <0.0001, Figure 5). The administration of rosuvastatin, regardless of the dosage used, decreased the SREBP-1 expression (compared to untreated HF group), although only the HF-R20 and HF-R40 groups exhibited no difference from the SC group.

## Discussion

The present model of metabolic syndrome, which is based on mice receiving a high-fat diet, is characterized by obesity, insulin resistance, glucose intolerance, dyslipidemia, and hepatic steatosis compatible with NAFLD. All of these changes were either attenuated or ameliorated by rosuvastatin in a dose-dependent manner. It is important to highlight that the dose of 10 mg/Kg mimics the usual clinical dose of humans following the dose translation proposed recently [27], whereas the largest doses are important to isolate the main effects in rodents once they present a more accelerated metabolism than humans and, hence, respond to bigger doses in the same extent humans respond to a small one [14]. Of note, the highest doses of rosuvastatin showed beneficial pleiotropic effects on the adipose tissue because it reduced the body mass, the fat mass and the adipocyte size and increased the subcutaneous fat mass (inguinal fat), as evidenced by the Visc:Sub ratio.

Excessive circulating free fatty acids (FFAs) are stored as ectopic fat in the muscle, liver and pancreas [28,29]. Thus, the visceral adipose tissue becomes mainly responsible for the development of the metabolic syndrome symptoms. In the present study, the HF diet caused weight gain, visceral adiposity, and adipocyte hypertrophy. The epididymal fat mass can ideally be used to assess the percentage of body fat because it correlates with the total body fat in humans [30]. A loss in body weight is usually correlated with both improvements in insulin sensitivity and reduced adipocyte size [31]. The administration of a rosuvastatin dose of 40 mg/kg/day decreased the adipocyte size and changed the pattern of fat storage because more fat was stored in the subcutaneous depot in the mice that received this treatment. Although the 10 and 20 mg/kg/day doses of rosuvastatin decreased the fat pad weight and the adipocyte size, these did not change the fat distribution between the visceral and subcutaneous depots. We did not expect the observed change in the adipose tissue biology; thus, further studies are necessary to obtain a better comprehension of the mechanisms that regulate the effect of rosuvastatin on adipose tissue.

Even though HF animals treated with rosuvastatin showed decreased fat pad masses and body masses than untreated HF animals, a progressive increase of feed efficiency was perceived in the treated groups. We hypothesize that effects of rosuvastatin upon lipid metabolism, adipose tissue remodeling, insulin resistance alleviation and NAFLD amelioration was so marked that even with a larger intake of energy, animals treated with 20 mg/Kg and 40 mg/Kg of rosuvastatin had a better body weight control than untreated and the ones treated with 10 mg/Kg of the drug.

The mechanisms that lead to NAFLD development vary and are associated with an unbalance of several cellular processes related to the signaling pathways of insulin, including the increased flow of FFA to the liver (lipolysis), the de novo lipogenesis of FFA, decreased beta-oxidation, mitochondrial dysfunction, oxidative stress, and endoplasmic reticulum stress [32]. Several evidences show that NAFLD development is associated with the amount of visceral fat, AST, ALT, total cholesterol, triglycerides, serum insulin and insulin resistance, which is assessed by the HOMA-IR.

The administration of rosuvastatin (20 mg/kg/day) improved the insulin sensitivity, decreased the liver steatosis and the body weight, and improved the circulating levels of cholesterol and triglycerides in mice fed a HF diet [14]. In the present study, the untreated HF mice exhibited increased levels of serum TG, TC and ALT and hepatic steatosis (micro and macrovesicular). Rosuvastatin reduced the steatosis in a dose-dependent manner, likely due to a decreased input of FFAs and an increased output of FFAs through increased beta-oxidation. Weight loss is directly correlated with the reduction of fat depots, which in turn reduces the release of FFAs [33].

Ob/ob mice that are fed polyunsaturated fatty acids (PUFAs) exhibit improved steatosis, reduced triglyceride storage in the liver, and the suppression of SREBP-1 expression [34]. In the present study, rosuvastatin had a dose-dependent effect on the SREBP-1 expression in the liver of mice fed the high-fat diet. SREBP-1 regulates not only the synthesis but also the storage in the liver of hepatic triglycerides [34]. Thus, from a therapeutic point of view, this enzyme might be a good target for the treatment of hepatic steatosis [35].

Some experimental evidence has shown that mitochondrial dysfunction has a crucial role in NAFLD genesis through the depletion of the mitochondrial DNA content, the reduction in the activity of the mitochondrial respiratory chain and deficient beta-oxidation [36,37]. In the present study, the livers from untreated HF mice had enlarged and morphologically unorganized mitochondria, as previously described [38], whereas the rosuvastatin-treated groups had numerous mitochondria that were morphologically well organized. Because this organelle is the main organelle responsible for beta-oxidation, these findings corroborate the hypothesis that increased beta-oxidation is essential for the prevention and/or reduction of hepatic damage in treated animals [39].

The analysis of the carbohydrate metabolism showed that the oral administration of glucose increases the release of insulin from pancreatic beta-cells [40], but the effect of statins on both carbohydrate metabolism and insulin resistance is still controversial [41].

Our main results agree with the latest evidence from epidemiological studies, reinforcing the importance of translational studies, which shed light on many pathways involved with the endpoints observed in humans. Recently, the randomized, placebo-controlled JUPITER (Justification for Use of statins in Prevention:an Intervention Trial Evaluating Rosuvastatin), a trial of rosuvastatin 20 mg, revealed a small risk of developing diabetes under statin therapy was limited to participants who had biochemical evidence of impaired fasting glucose or multiple components of metabolic syndrome prior to the treatment. Furthermore, the benefits of statin therapy surpassed the hazard of developing new onset diabetes both in participants with and without diabetes risk factors [16].

It is known that rosuvastatin has beneficial effects on the carbohydrate metabolism in non-diabetic patients with dyslipidemia compared with atorvastatin [42]. Fraulob et al. demonstrated that 20 mg of rosuvastatin ameliorates hepatic steatosis and glucose intolerance in a murine model of IR [14]. Corroborating these data, we found an improvement in the fasting glucose and glucose intolerance in mice treated for five weeks with rosuvastatin at a dose of 20 or 40 mg/kg/day. The analysis of the insulin resistance showed that similar doses of rosuvastatin and atorvastatin have the same effect on the HOMA-IR after eight weeks of treatment.

In conclusion, rosuvastatin improves glucose intolerance, insulin sensitivity and NAFLD in a dose-dependent manner and changes the fat distribution from visceral to subcutaneous fat depot in a mouse model of diet-induced obesity. Consequently, rosuvastatin therapy may be of great help to patients with metabolic syndrome because it has a wide range of beneficial pleiotropic effects.

### Limitations

This study presents some limitations. Firstly, the reduction in body weight gain in the HFR group-40 when compared with the other groups can be explained by a poor absorption of bowel function due to the high dosage of rosuvastatin (40 mg) used in this group. Even though intestinal modifications were not identified as the stools were normal without the presence of signs of intolerance and malabsorption. Secondly, a prospective follow-up study is needed to evaluate the effect of rosuvastatin treatment at different doses upon weight reduction and improvement of insulin resistance in mice, once some of the results found in the literature were in contrast with those found in human studies.

## Competing interests

This study was supported by the agencies CAPES (Coordination of Improvement of Higher Education Personnel, http://www.capes.gov.br) and Faperj (Rio de Janeiro Foundation for Research, http://www.faperj.br). The authors disclose that they have no financial interest or commercial sponsors for this work.

## Authors’ contributions

RNF and VNR carried out the western blot analysis, liver stereology and RIAs, performed the statistical analysis and drafted the paper. VS-M made substantial contributions to the study and was involved in revising the paper. CAMdL made substantial contributions to the research conception and design and was involved in revising the papert. JJdC was critically involved in writing, revising, drafting the paper and has given final approval of the version of the paper to be published. All authors read and approved the final manuscript.
